# The upper cervical spinal cord in ALS assessed by cross-sectional and longitudinal 3T MRI

**DOI:** 10.1038/s41598-020-58687-z

**Published:** 2020-02-04

**Authors:** Thomas Wimmer, Frank Schreiber, Nathalie Hensiek, Cornelia Garz, Jörn Kaufmann, Judith Machts, Susanne Vogt, Johannes Prudlo, Reinhard Dengler, Susanne Petri, Hans-Jochen Heinze, Peter J Nestor, Stefan Vielhaber, Stefanie Schreiber

**Affiliations:** 1 Department of Neurology, Otto-von-Guericke University Magdeburg, Magdeburg, Germany; 2 German Center for Neurodegenerative Diseases, Magdeburg Site, Magdeburg, Germany; 3 Leibniz Institute for Neurobiology (LIN), Magdeburg, Germany; 4 Department of Neurology, University Medical School Rostock, Rostock, Germany; 5 German Center for Neurodegenerative Diseases, Rostock Site, Rostock, Germany; 6 Department of Neurology, Hannover Medical School, Hannover, Germany; 7 Center for Behavioral Brain Sciences (CBBS), Magdeburg, Germany; 8 Queensland Brain Institute, The University of Queensland, Brisbane, Australia; 9 Mater Hospital, Brisbane, Australia

**Keywords:** Amyotrophic lateral sclerosis, Neuromuscular disease

## Abstract

The upper cervical spinal cord is measured in a large longitudinal amyotrophic lateral sclerosis (ALS) cohort to evaluate its role as a biomarker. Specifically, the cervical spinal cord´s cross-sectional area (CSA) in plane of the segments C1–C3 was measured semi-automatically with T1-weighted 3T MRI sequences in 158 ALS patients and 86 controls. Six-month longitudinal follow-up MRI scans were analyzed in 103 patients. Compared to controls, in ALS there was a significant mean spinal cord atrophy (63.8 mm² vs. 60.8 mm², p = 0.001) which showed a trend towards worsening over time (mean spinal cord CSA decrease from 61.4 mm² to 60.6 mm² after 6 months, p = 0.06). Findings were most pronounced in the caudal segments of the upper cervical spinal cord and in limb-onset ALS. Baseline CSA was related to the revised ALS functional rating scale, disease duration, precentral gyrus thickness and total brain gray matter volume. In conclusion, spinal cord atrophy as assessed in brain MRIs in ALS patients mirrors the extent of overall neurodegeneration and parallels disease severity.

## Introduction

Spinal cord involvement is a well-known and prominent feature of amyotrophic lateral sclerosis (ALS), that reflects both anterior horn cell (“amyotrophy”) and pyramidal tract degeneration/sclerosis of the lateral columns (“lateral sclerosis”) – which has been highlighted since Charcot’s pathology studies in 1865^1^. Autopsy studies correspondingly reveal mild to marked loss of motor neurons in around 80% of the ALS patients which can affect the anterior horn of the entire spinal cord from the level of C2 to the level of its caudal sacral segments^2^. Anterior horn cell loss is furthermore closely associated with large myelinated fiber degeneration of the pyramidal tract^2–4^.

*In-vivo* studies applying magnetic resonance imaging (MRI) correspondingly display a reduction of the spinal cord cross-sectional area (CSA) in ALS, i.e. showing spinal cord atrophy together with pyramidal tract integrity loss in diffusion tensor imaging (DTI)^5–7^.

Controversies remain, however, on the relationship of *in-vivo* MRI spinal cord atrophy to clinical features and biomarkers in ALS. While some studies reported an association between CSA reduction and worse motor performance or longer disease duration^8,9^, others failed to find such a clinical correlation^10,11^. Conflicting or negative results might stem from small sample sizes in that most studies included less than 30 patients^8,9,12^. Additionally, only a few ALS studies thus far took advantage of the capability of spinal cord *in-vivo* MRI to monitor CSA evolution over time^5,12,13^.

In consideration of these uncertainties, we conducted a retrospective analysis of the upper cervical spinal cord within brain MRIs that were acquired during a large cross-sectional (N = 158) and longitudinal (N = 103) multicenter study to understand how *in-vivo* spinal cord atrophy relates to clinical features, other biomarkers, and, how it evolves over time in ALS.

## Material and Methods

### Sample

This is a retrospective study of 3T brain MRI scans of ALS patients acquired between 04/2011 and 01/2014 and controls scanned between 09/2013 and 03/2014. Data analysis took place between 01/2017 and 12/2018. The cohort comprised N = 158 ALS patients recruited at the neuromuscular outpatient clinics of the Departments of Neurology at Otto-von-Guericke University, Magdeburg; Hannover Medical School, Hannover; and University Medical Center, Rostock, Germany. ALS diagnosis was based on the El Escorial criteria and its revisions^14–16^. Classification of clinical phenotypes along the ALS disease spectrum was in accordance with operational definitions and comprised classic, lower motor neuron dominant (LMND) and upper motor neuron dominant (UMND) ALS^17^.

In short, all patients with LMND ALS had clinical and electrophysiological evidence of sporadic progressive pure LMN involvement in one or more regions without clinical signs of UMN dysfunction. LMN involvement must be the predominant finding for at least 12 months after the symptom onset. Other LMN diseases, such as multifocal motor neuropathy, spinal muscular atrophy, monomelic amyotrophy, Kennedy disease, and post-polio syndrome were excluded by extensive clinical and laboratory examinations^18,19^.

In UMND ALS patients, LMN signs were restricted to only one neuraxis level (bulbar, cervical, or lumbosacral), and electromyography (EMG) abnormalities were limited to sparse fibrillation potentials/positive sharp waves or minor enlargement of motor unit potentials in one or at most two muscles^20,21^ for at least 12 months after symptoms onset.

We also included patients with primary lateral sclerosis (PLS), a syndrome of pure upper motor neuron involvement that has been considered part of the motor neuron disease spectrum^22,23^. The diagnostic criteria for PLS required only UMN signs on examination at the time of baseline MRI^24^. Based on those criteria, at this time PLS diagnosis has been suspected in N = 9 patients, but N = 4 of them still not fulfilled the demand of a period of at least 4 years in which only UMN signs remained on examination^24^. At the time of data analysis, which took place some years after baseline MRI (please see above), all of those N = 4 patients with initially suspected PLS fulfilled the diagnostic criteria for PLS in that there were only UMN signs for a period of at least 4 years. We thus considered all N = 9 patients to suffer from PLS. The following were rigorously excluded from the PLS category: patients with clinical or EMG signs of LMN involvement; those with a disease that could mimic motor neuron disease; a family history of spastic paraparesis/tetraparesis; mutations of genes related to hereditary spastic paraplegia (SPG3A, SPG4. SPG7, SPG10, and SPG11^25^); and those with symptom onset before an age of 40 years.

N = 4 ALS patients fulfilled the diagnostic criteria for behavioral variant frontotemporal dementia^26^.

The study was approved by the *Ethik-Kommission der Otto-von-Guericke-Universität in Magdeburg*, the *Ethik-Kommission der Medizinischen Hochschule Hannover* and the *Ethikkommission an der Medizinischen Fakultät der Universität Rostock* (Approval No. 150/09, No. 07/17 and No. 16/17), and all subjects gave written informed consent. All methods were performed in accordance with the relevant guidelines and regulations.

#### Cross-sectional data ALS sample

As part of an ongoing multicenter study, all patients underwent 3T MRI of the brain (N = 141 in Magdeburg, N = 17 in Rostock, please see below), which also included the upper portion of the cervical spinal cord. Overall motor function was assessed by applying the revised ALS functional rating scale (ALSFRS-R) at baseline. Baseline disease duration was defined as the timespan between symptom onset and the first 3T MRI.

Ventilation status was available in N = 138 patients (87%), and of those, 16 (10%) were using non-invasive ventilation (NIV).

Criteria for NIV comprised dyspnea, a forced vital capacity of < 75% as predicted and/or daytime hypercapnia (pCO_2_ ≥ 45 mmHg)^27,28^.

In a subset of N = 63 ALS cases (40%), genetic testing was performed for superoxide dismutase 1 protein (SOD1) and chromosome 9 open reading frame 72 protein (C9orf72) gene mutations.

There were no differences between the ALS patients who had genetic data available and those who did not with regard to demographics (age, sex, height, weight) and clinical variables (baseline ALSFRS-R, baseline disease duration, onset site, clinical phenotype; data not shown).

#### Longitudinal data ALS sample

Of the 158 ALS patients, N = 103 cases (65%) underwent a second 3T MRI of the brain and upper cervical spinal cord and a second assessment of their motor function, i.e. ALSFRS-R, after 6 months. The baseline ALSFRS-R score of those who had longitudinal imaging was significantly better than that of the patients who only had a single scan (39 vs. 36 points, t(84) = −2.6, p = 0.01; independent-samples t-test). This is not unexpected because the less severe patients at baseline were more likely to undergo repeated imaging at 6-month follow-up. There were no further differences between patients with or without follow-up MRI with regard to their demographics (age, sex, height, weight) or clinical variables (baseline disease duration, onset site, clinical phenotype; data not shown).

#### Controls

For group comparisons, a community-based cohort of N = 86 participants recruited through public advertisements in Magdeburg (N = 69) and Rostock (N = 17), underwent a baseline 3T MRI of the brain and the upper cervical spinal cord using the same MRI protocol. None of the control subjects suffered from neuromuscular disorders, i.e. peripheral neuropathies, muscle or motor neuron diseases, nor from any brain injury, epilepsy or major psychiatric disease, nor did they display any specific abnormalities on the neurological exam^29,30^.

### 3T MRI of the brain and the upper cervical spinal cord

At both imaging sites (Magdeburg and Rostock) cerebral 3D-MPRAGE (magnetization-prepared rapid gradient-echo) images were acquired on a MAGNETOM Verio 3T MRI scanner (Siemens, Erlangen, Germany) using a 32-channel head coil applying exactly the same MRI protocol: TE (echo time) = 4.82 ms, TR (repetition time) = 2500 ms, TI (inversion time) = 1100 ms, flip angle = 7°, voxel size = 1 × 1 × 1 mm^3^, matrix = 256 × 256 × 192. MRI data acquisition time was 52 minutes.

### Determination of the cervical spinal cord area

**Figure 1:**
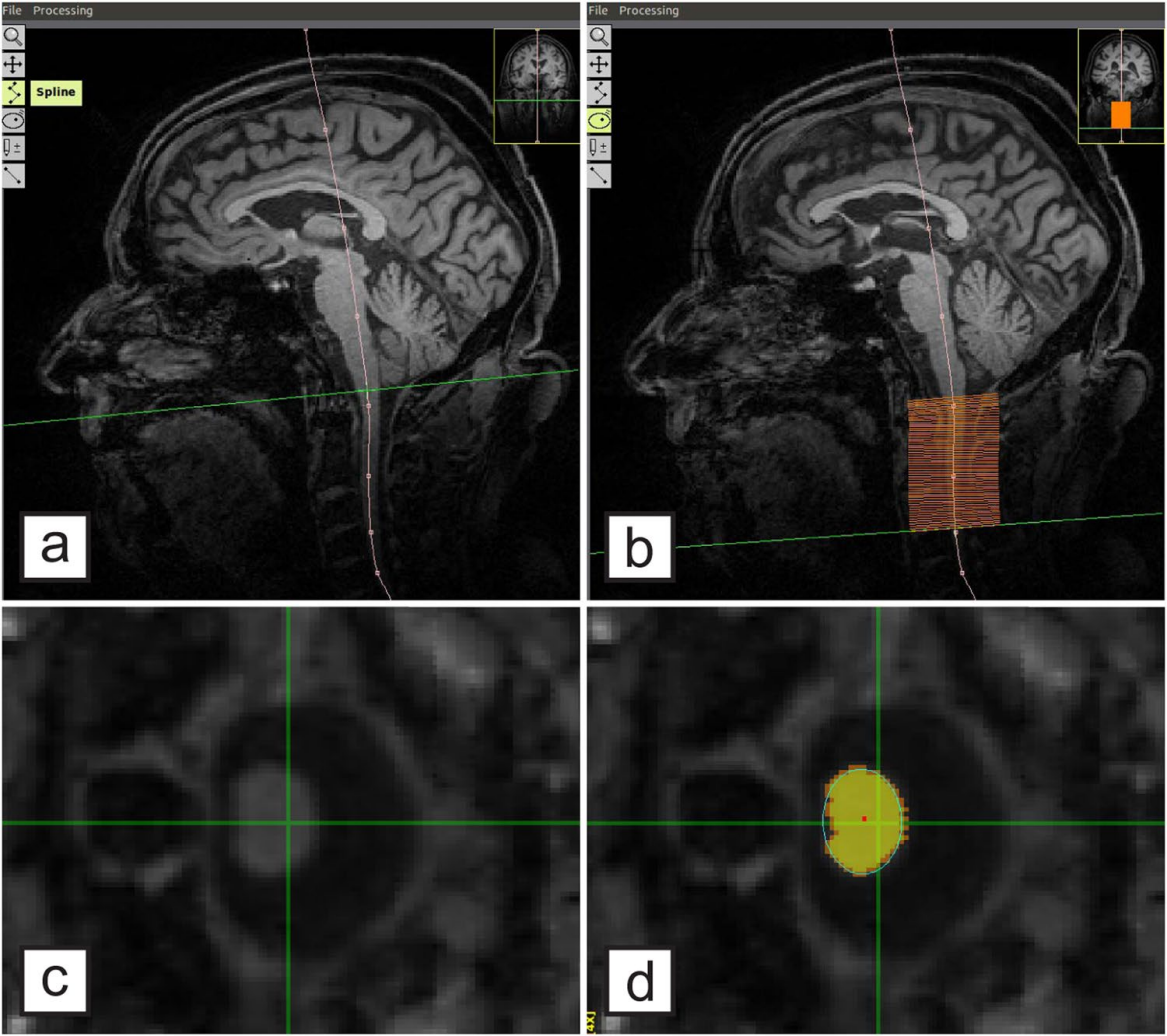
Measurement of the cervical spinal cord cross-sectional area (CSA) applying SpineSeg. **(a)** demonstrates the cerebral 3T MRI, covering the upper segments of the cervical spinal cord, in a sagittal view. A guiding curve (pink) was placed along the spinal cord in the coronal and sagittal plane; the image is consequently resampled into perpendicular slices. Within the slices from the dens axis until the second intervertebral disk **(b)**, the cervical spinal CSA segmentation was derived using a semi-automated tree pruning approach. Transversal view of the spinal cord is demonstrated before **(c)** and after **(d)** segmentation, respectively. **(d)** Seed in red, segmentation in yellow.

All scans were visually inspected to exclude spondylotic myelopathy. The cervical spinal cord CSA was determined with the freely available software SpineSeg, developed by Bergo *et al*.^31^. The contour of the spinal cord in the sagittal and coronal plane was tracked using a spline approximation and the volume was subsequently resampled into slices perpendicular to this guiding curve. The spinal cord was then semi-automatically segmented in each of these resampled axial slices using a tree pruning approach^32^ starting from the upper boundary of the dens axis to the middle of the second intervertebral disk, covering the upper cervical spinal cord segments C1, C2 and C3 **(**Fig. 1**)**. Scans in which the delineation at C3 was not possible, either because of a decreased contrast resulting from the intensity attenuation towards the inferior edge of the field of view or because C3 was not covered, were excluded from the study. For baseline MRI, the scans of N = 2 controls had to be excluded; for follow-up MRI, the scans of N = 5 ALS patients had to be excluded. The mean number of slices per subject did not differ between patients and controls (t(240) = −0.8, p = 0.4).

For group comparison, the mean of each subject’s CSA of all available spinal cord slices was calculated. All measures were performed twice by one researcher (TW) blinded to the clinical diagnosis. Corresponding intra-class correlation coefficient (ICC) was excellent (0.99). In 33 randomly chosen ALS patients and 26 controls, CSA measurements were repeated by a second researcher (FS), with an excellent inter-rater reliability (ICC = 0.98).

For analysis of distribution along the examined spinal cord length, five intermediate positions between dens axis and the center of the first intervertebral disk and two between first and second intervertebral disk were calculated. The slices closest to these chosen positions were used for comparison, creating an approximately equidistant sampling with a median of 5 [4–6] mm spacing.

### Cortical Thickness and brain volumetric measures

To relate the cervical spinal cord CSA to structural measures of cerebral motor and extra-motor involvement, for each patient cortical thickness of the bilateral precentral gyrus was obtained from the native-space MPRAGE scans using the automated FreeSurfer 6.0 parcellation^33^. In both ALS and controls the right precentral gyrus was significantly thinner than the left (for ALS: 2.33 mm vs 2.43 mm, Z(11232) = 8.8, p < 0.001; for controls: 2.4 mm vs 2.49, Z(3127) = −6.6, p < 0.001; Wilcoxon signed-rank test). For statistical analysis the right and left motor cortex were thus considered separately. Total cerebral gray matter volume (GMV) normalized for head size was further estimated using the SIENAX algorithm from the SIENA-package of FMRIB Software Library (FSL) v5.0.

### Statistics

Statistical analysis was conducted using IBM SPSS Statistics (Version 22.0).

Normal distribution of the data was assessed with a Shapiro-Wilk Test.

Group comparisons of the baseline mean cervical spinal cord CSA between (i) ALS vs. controls, (ii) limb- vs. bulbar-onset ALS, (iii) selected clinical phenotypes (e.g. classic ALS) vs. the remainder, (iv) sporadic vs. familial ALS and (v) non-ventilated patients vs. those requiring assisted ventilation were conducted using an analysis of variance (ANOVA). Additional group comparisons between ALS vs. controls along the length of the cervical spinal cord were performed, also applying an ANOVA. Baseline cervical spinal cord CSA depended on baseline age (see Results), and thus all models were adjusted for baseline age.

The relationship between mean spinal cord CSA and the patients’ and controls’ demographics (age, sex, height, weight); the patients’ clinical features (baseline ALSFRS-R and its sub-scores for bulbar fine motor, gross motor, respiratory function, baseline disease duration) and imaging data (motor cortex thickness, GMV) were assessed applying bivariate correlations and a χ² test.

Differences between baseline and follow-up cervical mean spinal cord CSA were calculated for ALS and for limb- and bulbar-onset ALS separately, applying a repeated measure ANOVA. Further, another repeated measure ANOVA was conducted to calculate any differences between baseline and follow-up ALSFRS-R. For ALS, additionally, differences between baseline and follow-up local CSA along the spinal cord were computed, also using a repeated measure ANOVA. All models were adjusted for baseline age.

P-values ≤ 0.05 were deemed statistically significant.

## Results

### Sample

Demographics of the whole cohort and the clinical data of the ALS patients are given in Table 1. There were no group differences between ALS and controls with respect to age, sex, height and weight.

**Table 1:** Demographics and baseline clinical data of the sample under consideration.

	ALS N = 158	Controls N = 86	Statistics
Age in years	61 (31–82)	62 (33–82)	Z = −0.4, p = 0.7^a^
Male sex, n (%)	100 (63)	52 (62)	χ²(1) = 0.1, p = 0.8^b^
Height in cm	172 [10]	174 [9]	t(140) = −0.7, p = 0.5^c^
Weight in kg	75 [14]	79 [14]	t(126) = −1.0, p = 0.3^c^
Definite ALS^1^ / Probable ALS^1^ / Possible ALS^1^ / Suspected ALS^1^ / PLS, n (%)	12 (8) / 66 (42) / 43 (27) / 28 (18) / 9 (6)	N/A	N/A
Classic ALS / LMND ALS / UMND ALS / PLS, n (%)	108 (68) / 27 (17) / 14 (9) / 9 (6)	N/A	N/A
Limb- / bulbar-onset, n (%)	110 (71) / 45 (29)*	N/A	N/A
Disease duration (months)	16 (3–272)	N/A	N/A
ALSFRS-R total score	39 (14–48)	N/A	N/A
Sporadic / familial ALS, n (%)	49 (78) / 14 (22)**	N/A	N/A
No NIV / NIV, n (%)	122 (77) / 16 (10)***	N/A	N/A

Unless otherwise reported, mean [SD] or median (range) is given. For group comparisons a Mann-Whitney U test^a^, χ² test^b^, or an independent-samples t test^c^ was conducted.

ALS, amyotrophic lateral sclerosis; ALSFRS-R, revised ALS functional rating scale; LMND, lower motor neuron dominant; N, number; N/A, not applicable; NIV, non-invasive ventilation; PLS, primary lateral sclerosis; UMND, upper motor neuron dominant. ^1^, according to the El Escorial criteria and its revisions^14–16^. Data were missing in *3, **95 and ***20 patients, respectively. **Of the 14 familial ALS cases, 7 (11%) had mutations in the SOD1 gene and 7 (11%) in the C9orf72 gene. P-values ≤ 0.05 were deemed statistically significant.

### Cross-sectional data

**Figure 2:**
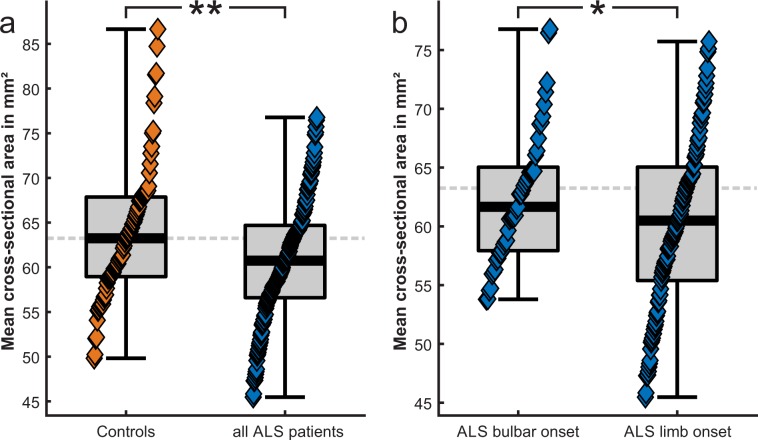
Quantile function of baseline mean cervical spinal cord cross-sectional area in ALS and controls. **(a)** CSA in ALS was significantly smaller than in controls. **(b)** CSA was further significantly smaller in limb- compared to bulbar-onset ALS. *p ≤ 0.05, **p ≤ 0.001 (all statistical models are adjusted for baseline age).

**Figure 3:**
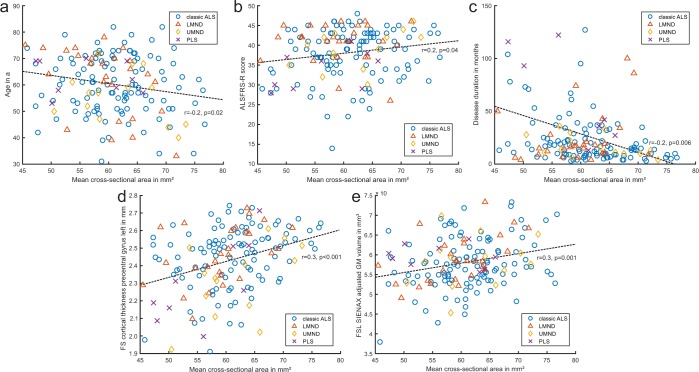
Relationship between baseline mean cervical spinal cord cross-sectional area (CSA) and various variables in ALS. CSA relates to baseline **(a)** age, **(b)** total revised ALS functional rating scale (ALSFRS-R), **(c)** disease duration, **(d)** cortical thickness of left precentral gyrus, **(e)** total adjusted gray matter (GM) volume. Some patients labelled PLS did not fulfill the demand of a period of at least 4 years with UMN signs only at the time of 3T MRI, but would go on to be diagnosed with PLS after the study’s conclusion. FS, Freesurfer; FSL, FMRIB Software Library; LMND, lower motor neuron dominant ALS; UMND, upper motor neuron dominant ALS; PLS, primary lateral sclerosis. P-values ≤ 0.05 were deemed statistically significant.

For the whole cohort, baseline mean spinal cord CSA was not normally distributed (D[242] = 1.0, p = 0.03). When considering ALS patients and controls separately, mean CSA was normally distributed in ALS (D[158] = 1.0, p = 0.5), but not in controls (D[84] = 1.0, p = 0.003) **(**Fig. 2a). Considering the whole cohort, mean CSA was related to baseline age (r = −0.2, p = 0.003) in that older age was associated with a smaller spinal cord CSA **(**Fig. 3a**)**. Mean CSA was not related to sex, height and weight. These results remained unchanged when considering ALS and controls separately.

In ALS compared to controls, mean CSA was significantly smaller (60.8 mm² vs. 63.8 mm², F(1,240) = 10.7, p = 0.001; Fig. 2a).

**Figure 4:**
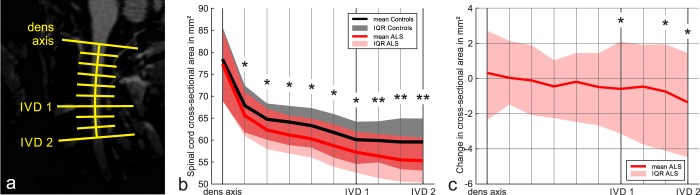
Cross-sectional area along the studied upper cervical spinal cord length in ALS and controls. For analysis of CSA distribution along the spinal cord, five intermediate positions between dens axis and the center of the first intervertebral disk (IVD) and two between first and second IVD were calculated, as indicated in **(a)**. The cross-sectional area in these positions is compared between ALS and controls in **(b)**, displaying means for ALS and controls as well as the interquartile range (IQR) for both groups. Significantly smaller CSA values were found in ALS in all positions below the dens axis. The longitudinal change in CSA along the spinal cord in ALS in shown in **(c)**, indicating that significant CSA changes are only found in the more caudal areas between IVD 1 and IVD 2. *p ≤ 0.05, **p ≤ 0.001 (all statistical models are adjusted for baseline age).

The results of the CSA analysis along the length of the upper cervical spinal cord are displayed in Fig. 4a,b. At the dens axis level there were no significant group differences between ALS and controls, while all of the more caudal points down to the first intervertebral disk revealed a group difference with p < 0.05. The points of the first intervertebral disk until the second intervertebral disk revealed more significant differences with p < 0.001.

Furthermore, ALS patients with limb-compared to bulbar-onset had a significantly smaller baseline mean spinal cord CSA (60.2 mm² vs. 62.5 mm², F(1,153) = 5.4, p = 0.02; Fig. 2b). Limb- and bulbar-onset ALS patients did not differ significantly with respect to age, disease duration or disease severity (according to baseline ALSFRS-R); group differences were thus not driven by those variables.

There were, however, no group differences for the mean spinal cord CSA when considering the ALS phenotypes, sporadic against familial ALS or non-ventilated against ventilated patients. Detailed results of all group comparisons are given in Table 2.

**Table 2:** Group comparisons of the baseline cervical spinal cord cross-sectional area.

Group	N	CSA in mm^2^	Statistics
ALS	158	61 [7]	F(1,240) = 10.7, **p = 0.001**
Controls	86	64 [7]	
Limb-onset ALS	110	60 [7]	F(1,153) = 5.4, **p = 0.02**
Bulbar-onset ALS	45	62 [6]	
Classic ALS	108	61 [7]	F(3,154) < 1.7, p > 0.2
LMND ALS	27	59 [6]	
UMND ALS	14	62 [7]	
PLS	9	56 [7]	
Sporadic ALS	49	61 [7]	F(1,61) = 1, p = 0.4
Familial ALS	14	63 [8]	
No NIV	122	61 [7]	F(1,136) = 0.9, p = 0.4
NIV	16	61 [6]	

Mean [SD] of the baseline cervical spinal cord cross-sectional area (CSA) is given. ALS, amyotrophic lateral sclerosis; LMND, lower motor neuron dominant; N, number; NIV, non-invasive ventilation; PLS, primary lateral sclerosis; UMND, upper motor neuron dominant. P-values ≤ 0.05 were deemed statistically significant. Statistical models are adjusted for baseline age.

In the ALS sample, mean CSA was related to clinical variables, i.e. positively correlated with baseline ALSFRS-R total score (r = 0.2, p = 0.04); its gross motor function subscore (r = 0.2, p = 0.03); and negatively correlated with disease duration (r = −0.3, p < 0.001) **(**Fig. 3b,c). It was also positively correlated with precentral gyrus thickness (left r = 0.3, p < 0.001; right r = 0.2, p = 0.04) and GMV (r = 0.2, p = 0.02) (Fig. 3d,e). In summary, a larger mean spinal cord CSA was related to shorter disease duration, higher functional scoring, greater motor cortex thickness and GMV.

### Longitudinal data

**Figure 5:**
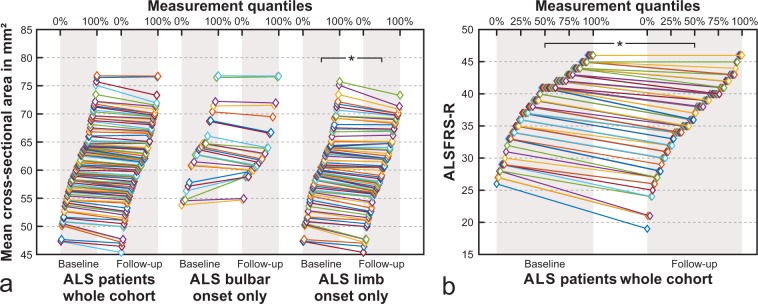
Baseline and follow-up mean cervical spinal cord cross-sectional area and ALSFRS-R in ALS. Markers are spread out to display measurement quantiles, as given in the top axis. *p ≤ 0.05 (all statistical models are adjusted for baseline age).

Longitudinal analysis revealed a trend towards a significant decline of the cervical spinal cord mean CSA from baseline to follow-up in ALS (61.4 vs. 60.6 mm², F(1,101) = 3.7, p = 0.06) (Fig. 5a). As displayed in Fig. 4c, CSA decline was most pronounced in the more caudal parts of the upper cervical spinal cord towards the second intervertebral disk. When considering limb- vs. bulbar-onset patients separately, only those with limb-onset displayed a significant mean CSA decline (60.6 vs. 59.6 mm^2^, F(1,75) = 4.8, p = 0.03), while bulbar-onset patients did not (63.9 vs. 63.6 mm^2^, F(1,23) = 0.1, p = 0.7) **(**Fig. 5a**)**. Likewise, there was a significant decline of the ALSFRS-R from baseline to follow-up in ALS, with a larger effect size than the decline of the spinal cord mean CSA (39.1 vs. 35.8, F(1,99) = 15.9, p < 0.001) **(**Fig. 5b**)**. There was nevertheless a small effect-size correlation between ΔCSA and ΔALSFRS-R in ALS (bivariate correlation, r = 0.2, p = 0.03).

## Discussion

This large, multicenter study of the brain confirmed atrophy of the adjacent upper cervical spinal cord cross-sectional area and a trend towards its ongoing decline over 6 months in ALS, especially in the upper cervical spinal cord’s caudal segments and in limb-onset disease. Mean cervical spinal cord area related further to several clinical variables and measures, such as motor function, disease duration, motor cortex thickness and whole brain gray matter volume. *In-vivo* MRI upper cervical spinal cord atrophy assessed in a brain MRI can thus be considered a reliable biomarker that reflects disease severity. This highlights that measuring the upper spinal cord as part of the (routine) brain MRI scan is a valid marker in research studies.

Compared to most previous studies, our ALS cohort was nearly fivefold larger, allowing for subgroup comparisons along the motor neuron disease spectrum. The most severe cord atrophy was found in PLS in the present study, although this did not reach statistical significance, almost certainly because of insufficient power due to the small PLS subgroup size. We propose that upper spinal cord atrophy across the motor neuron disease spectrum is largely driven by corticospinal tract degeneration - a hypothesis that would explain the PLS finding in that this sub-group would not be expected to have significant anterior horn involvement. This is furthermore reinforced by the fact that the upper spinal cord is dominated by the white matter fraction (around 85% of the CSA), while it declines along lower cervical spinal cord levels (around 80%)^34^. However, more severe cord atrophy in PLS patients could additionally be explained by a longer disease duration than in ALS patients.

Upper cervical spinal cord atrophy could thus be considered to serve as a biomarker of upper motor neuron involvement, which by now is mainly assessed clinically depending on the experience of the physician. This could be one major role of cross-sectional and longitudinal spinal cord imaging despite its effect size of change is smaller than that of the longitudinal alteration of the clinical state of the ALS patients, i.e. of scoring their motor function applying the ALSFRS-R. Indeed, the ALSFRS-R is a composite score measuring the function of 4 different levels, comprising the patients’ bulbar state; its decline does thus not reflect the loss of any specific motor ability. The ALSFRS-R is further a measure that predominantly indicates the involvement of the lower motor neuron. The correlation between baseline spinal cord CSA and ALSFRS-R as well as the relationship between the decline of the two variables, however, reflects the common direction of imaging and clinical variables. Clinical trials should thus carefully take into account the, presumably, additive value of imaging and clinical measures in ALS.

Spinal cord atrophy and its ongoing decline was more pronounced in limb- compared to bulbar-onset patients confirming the results of one large autopsy study in ALS^2^. These findings are also consistent with a recent case-series that failed to identify significant cervical spinal cord atrophy in bulbar predominant ALS^11^. In addition, in bulbar-onset ALS spinal cord CSA did not decline within a period of 6 months. This resonates with the clinical observation that in bulbar predominant ALS spinal cord involvement takes place at later disease stages^2,35^, which has relevance when considering spinal cord atrophy as a monitor variable in clinical trials. One has, however, to consider some recruitment bias in that patients with severe bulbar involvement that cannot lie down for a longer period did not undergo MRI, especially longitudinal scanning. In that instance, it has to be considered remarkable that our cohort included 10% ALS patients with NIV, which are suffering from the same restrictions and are thus otherwise rarely included into MRI studies. The exclusion of patients with severe bulbar involvement might indeed have shifted our cohort towards (bulbar-onset) patients with a less rapidly progressive overall disease course and thus, less severe spinal cord involvement.

In the present study, reduction of mean upper cervical spinal cord CSA was around 5% in ALS compared to controls. Atrophy was greater in all other thus far conducted upper cervical spinal cord studies ranging between 10% to 17%^5,8,10,11,13^. All of these studies focused their measurements at the slice in the center of the intervertebral disc between C2 and C3 and few adjacent slices. In contrast, this study covered the whole length of the upper cervical spinal cord segments C1, C2 and C3 to the middle of the second intervertebral disk, to establish the atrophy along this section. The non-uniform manifestation of CSA reduction along the evaluated length results in lower mean atrophy estimates, despite the locally higher CSA reduction found in more caudal segments. Moreover, our ALS sample was at an earlier disease stage, reflected by a shorter median baseline disease duration of 16 months and relatively preserved motor function (median baseline ALSFRS of 39 points), compared to all previous cohorts. Accordingly, the ALS study reporting the greatest cord atrophy (17%) had the longest disease duration (44 months) and a lower ALSFRS-R (35 points)^11^. However, differences in measurement methods, MRI sequences (T1, T2) and field strengths (1.5 T vs. 3T) may have also played a role. Furthermore, the large white matter fraction of the upper cervical spinal cord (see above) should give rise to worse atrophy in samples with greater proportions of PLS and UMND ALS. As only one previous study reported ALS phenotypes^11^, it remains speculation as to whether this might have contributed to differences between studies.

A major strength of our study was the availability of longitudinal MRI datasets. The spinal cord CSA atrophy rate in our ALS sample was −1.7% over 6 months, which is somewhat smaller than that of previous studies showing rates of −2.5% (over 9 months) or −4.4% (over 8 months)^5,13^. Importantly, our longitudinal sample was also approximately four- to five times larger than that of past studies, suggesting the present atrophy rate is, arguably, a more robust estimate of reality. Different timespans, disease stages and various proportions of limb-onset ALS may have contributed to the differences as well. Overall, the results indicate that the upper spinal cord CSA might potentially be helpful to track ongoing disease even over a six-month interval but only provided the cohort is substantial (well in excess of 100 patients).

Turning to technical considerations, cross-sectional area is the most often used measure to assess spinal cord atrophy with excellent reliability^31^. Other measures such as the anterior-posterior diameter^36^ or spinal cord eccentricity^8,13^ are not useful biomarkers being insensitive to cord atrophy in ALS.

A range of methods has been employed to measure cervical cord CSA in ALS studies. In our study, the CSA was assessed with a semi-automated method (SpineSeg)^31^ which was highly reliable (both intra- and inter-rater)^8,13,31^. It took 10 to 15 minutes to process a subject, which is comparable to other semi-automated methods, e.g. Losseff’s approach^37^. Given the high reliability of semi-automated spinal cord CSA measures, new fully automated approaches could now be benchmarked against this approach.

Similar to several previous studies, we focused on the assessment of the upper part of the cervical spinal cord^8,13^. The main advantages of this approach are that it does not require additional MRI scans of the spinal cord, which are time consuming (potentially important given the difficulty many ALS patients have to lie flat for long periods); demand specific coils and dedicated sequences/protocols; need cardiac and respiratory gating; and are less standardized within multicenter studies. The upper cervical spinal cord can instead easily be included in an MRI brain acquisition and is, furthermore, useful in suspected limb-onset disease to exclude compressive myelopathy. One has, however, to take into account that – in a brain MRI - the upper part of the cervical spinal cord (i.e. especially C2 and C3 levels) is often at the limit of the field of view and its measure can be very variable between the acquisitions. The spinal cord area might thus often be difficult to be analyzed with a sufficient precision. Moreover, one has to keep in mind that the exploration of the lower parts of the cervical cord, although more technically challenging, is very relevant in ALS patients since lower motor neuron involvement is usually most pronounced at the C5 to Th1 level^2^.

## Conclusion

Our study demonstrated mean upper cervical spinal cord atrophy that was – on a trend-level - detectable within a six-month window, and, that was related to clinical measures and markers of lower and upper motor neuron pathology in ALS. Atrophy was most pronounced in the caudal upper cervical spinal cord segments and in limb- compared to bulbar-onset disease, but was found in all clinical phenotypes along the ALS disease spectrum. The results emphasize the potential role of *in-vivo* MRI upper cervical spinal cord cross-sectional area to serve as a valid quantifiable marker for ALS, perhaps also for the longitudinal monitoring of ongoing disease. Future studies should focus on the differences between gray and white matter atrophy not only in the different ALS phenotypes, but also by taking into account brain and spinal DTI parameters and their correlation with the cervical spinal cord area.

## Data Availability

All custom scripts and all relevant data of this study are available from the corresponding author upon reasonable request.
